# Long term clinical history of an Italian cohort of infantile onset Pompe disease treated with enzyme replacement therapy

**DOI:** 10.1186/s13023-018-0771-0

**Published:** 2018-02-08

**Authors:** Rossella Parini, Paola De Lorenzo, Andrea Dardis, Alberto Burlina, Alessandra Cassio, Paolo Cavarzere, Daniela Concolino, Roberto Della Casa, Federica Deodato, Maria Alice Donati, Agata Fiumara, Serena Gasperini, Francesca Menni, Veronica Pagliardini, Michele Sacchini, Marco Spada, Roberta Taurisano, Maria Grazia Valsecchi, Maja Di Rocco, Bruno Bembi

**Affiliations:** 1 Pediatric Rare Diseases Unit, Department of Pediatrics, MBBM Foundation, ATS Monza e Brianza, Via Pergolesi 33, 20900 Monza, Italy; 2 Centre of Biostatistics for Clinical Epidemiology, School of Medicine and Surgery, University of Milano-Bicocca, Monza, Italy; 3 Centre for Rare Diseases, University Hospital Santa Maria della Misericordia, Udine, Italy; 4 Department for Women and Children’s Health, U.O.C. Inborn Metabolic Diseases, University Hospital, Padova, Italy; 5 Department of Pediatrics, University of Bologna, Bologna, Italy; 6 Department of Pediatrics, University Magna Graecia, Catanzaro, Italy; 7 Department of Translational Sciences, Pediatrics, University Federico II, Naples, Italy; 8 Division of Metabolism Bambino Gesù Children’s Hospital, Rome, Italy; 9 Department of Pediatrics, Meyer Children’s Hospital, Metabolic and Muscular Unit, University of Firenze, Florence, Italy; 10 Department of Clinical and Experimental Medicine, Metabolic Diseases, Pediatric Clinic, University of Catania, Catania, Italy; 11 Department of Pathophysiology and Transplantation, Pediatric Highly Intensive Care Unit, University of Milano, IRCCS Ca’ Granda Ospedale Maggiore Policlinico Foundation, Milan, Italy; 12 Department of Pediatrics, University of Torino, Torino, Italy; 13 Rare Diseases Unit, Pediatric Hospital Istituto Giannina Gaslini, Genoa, Italy

**Keywords:** Infantile onset Pompe disease, Alglucosidase alpha, ERT, Recombinant human GAA, rhGAA

## Abstract

**Background:**

Enzyme replacement therapy (ERT) has deeply modified the clinical history of Infantile Onset Pompe Disease (IOPD). However, its long-term effectiveness is still not completely defined. Available data shows a close relationship between clinical outcome and patients’ cross-reactive immunological status (CRIM), being CRIM-negative status a negative prognostic factor. At the same time limited data are available on the long-term treatment in CRIM-positive infants.

**Methods:**

A retrospective multicentre observational study was designed to analyse the long-term effectiveness of ERT in IOPD. Thirteen Italian centres spread throughout the country were involved and a cohort of 28 patients (15 females, 13 males, born in the period: February 2002–January 2013) was enrolled. IOPD diagnosis was based on clinical symptoms, enzymatic and molecular analysis. All patients received ERT within the first year of life. Clinical, laboratory, and functional data (motor, cardiac and respiratory) were collected and followed for a median period of 71 months (5 years 11 months).

**Results:**

Median age at onset, diagnosis and start of ERT were 2, 3 and 4 months, respectively. CRIM status was available for 24/28 patients: 17/24 (71%) were CRIM-positive. Nineteen patients (67%) survived > 2 years: 4 were CRIM-negative, 14 CRIM-positive and one unknown. Six patients (5 CRIM-positive and one unknown) never needed ventilation support (21,4%) and seven (6 CRIM-positive and one unknown: 25%) developed independent ambulation although one subsequently lost this function. Brain imaging study was performed in 6 patients and showed peri-ventricular white matter abnormalities in all of them. Clinical follow-up confirmed the better prognosis for CRIM-positive patients, though a slow, progressive worsening of motor and/or respiratory functions was detected in 8 patients.

**Conclusions:**

These data are the result of the longest independent retrospective study on ERT in IOPD reported so far outside clinical trials. The data obtained confirmed the better outcome of the CRIM-positive patients but at the same time, showed the inability of the current therapeutic approach to reverse or stabilize the disease progression. The results also evidenced the involvement of central nervous system in Pompe disease. To better understand the disease clinical history and to improve treatment efficacy larger multicentre studies are needed as well as the development of new therapeutic approaches.

## Background

Enzyme replacement therapy (ERT) with recombinant human acid α-glucosidase (rhGAA) for Infantile Onset Pompe Disease (IOPD) (OMIM #232300) became commercially available in 2006 on the basis of two pivotal multicentre studies [1, 2]. These first studies demonstrated the improvement of cardiomyopathy and a prolonged survival in 26 treated infants and the progress of motor function milestones in some of them.

Further studies indicated that the age of ERT start was critical to obtain a better therapeutic result [3, 4]**.** Furthermore, data of literature showed an inverse correlation between the titer of anti-rhGAA IgG antibodies and the clinical outcome [5]. Patients with high titer were mainly cross-reactive immunologic material negative (CRIM-negative) and were not able to synthesize any kind of GAA protein, in contrast to CRIM-positive patients who produced a non-functional form of GAA [5]. In an attempt of suppressing anti-rhGAA IgG production, immunosuppression and ERT-naïve immunomodulation protocols have also been used in CRIM-negative patients with apparent success [6–9].

Recently, the efficacy of very early treatment (≤1 months of age) in CRIM-positive patients was reported in a number of studies from Taiwan, where a newborn screening program for Pompe disease has been performed since 2008 [10, 11]. By contrast a more variable and unpredictable outcome has been reported in CRIM-positive IOPD patients, who were diagnosed by clinical symptoms and received long-term ERT outside clinical trials[12–15]. Indeed, many patients have progressively lost the reached motor milestones and showed an impairment of respiratory function with the need of ventilation support [12–15]. Moreover, two studies on a limited number of CRIM-positive patients suggest that in the long-term some patients may probably benefit from a higher ERT dosage than presently recommended [16, 17].

Finally, a progressive white matter damage, presumably related to brain glycogen accumulation, has been shown in a number of patients [18–21]. This under-recognized problem adds up to other still unanswered questions regarding the optimal treatment approach for this rare disease.

In the present paper, we document the long-term outcome of 28 Italian IOPD patients treated with ERT with a median follow-up time of 6 years. The data reported here significantly contribute to improve the knowledge of long-term ERT outcomes of IOPD patients.

## Methods

A retrospective multicentre observational study was designed to analyse the long-term clinical history of a cohort of IOPD treated with ERT. The study involved 13 Italian centres and enrolled overall 28 patients (15 females, 13 males) born in the period: February 2002–January 2013. Patients inclusion criteria were: a) confirmed diagnosis of IOPD, based on clinical symptoms, enzyme and molecular analysis; b) to receive ERT.

Collected clinical and functional data included: age at diagnosis, age at ERT start, signs and symptoms at disease onset and during the follow-up visits; achieved motor functions; heart hypertrophy and ejection fraction normalization (yes/no/partially); respiratory function (need of ventilatory support); speech development and language intelligibility; hearing function; feeding impairment. Laboratory parameters included: transaminases, creatine kinase and IgG antibodies to rh-GAA; residual GAA activity (in lymphocytes, fibroblasts or muscle tissue); GAA mutation profile; CRIM status. Magnetic resonance imaging (MRI) data of the brain were analysed when available.

All patients received alglucosidase-alfa treatment within the first 12 months of age (8 of them ≤3 months). Baseline ERT dosage was 20 mg/Kg/every other week (eow) in 26 patients, while the other 2 (patients: 27 and 28), who had participated in a clinical trial [2, 4] received 40 mg/Kg/eow. Due to poor clinical outcome or to infusion associated reactions (IAR), ERT dosage was modified in course of follow-up in 7 patients (Table 1). Informed consent to data collection was obtained for all patients by parents or the legal representative.

**Table 1:** Genotypes, cross reactive immunological material (CRIM) status, ages of onset of symptoms, diagnosis and starting ERT, immunological data, Infusion associated reactions and ERT dosing of the 28 Pompe patients

Patient ID/gender	Genotype	Predicted mutations severity^a^	CRIM	Age of onset signs and symptoms^b^	Age diagnosis^b^	Age starting ERT^b^	Anti-rh GGA antibodies maximum titer	Immunomodulation	Severe IAR	ERT present or last dosing
1/M	NA	–	NA	1	1	2	NA	No	No	1
2/F	NA	–	NA	2	4	4	NA	No	Yes	1
3/F	c.[1833_1839del;1846G > T; 1847_1848insT]; [c1124G > T]	Very severe/potentially less severe	P(E)	1	5	5	NA	No	No	1
4/M	NA	–	NA	3	4	4	NA	No	No	1
5/F	c.[236_246 del];. [236_246 del]	Very severe	Neg (E)	1	1	2	1:25.600	No	Yes	1
6/F	c.[742delC];[c.896 T > C]	Very severe/potentially less severe	P(E)	4	6	10	NA	No	No	1
7/F	c.[2481 + 102_2646 + 31del]; [2481 + 102_2646 + 31del]	Very severe	Neg(E)	6	8	8	NA	No	No	1
8/F	c.[525delT];[c.670C > T]	Very severe/potentially less severe	P(E)	1	9	10	NA	No	No	1
9/M	c.[1497G > A];[1497G > A]	Very severe	Neg	birth	5 days	1	1:102.400	No	No	1
10/M	c.[1833_1839del;1846G > T; 1847_1848insT]; [1833_1839del;1846G > T; 1847_1848insT]	Very severe	Neg (E)	2	7	7	1:25.600	Yes 14 m [7]	Yes	1
11/M	c.[1942G > A]; [2646 + 2 T > A]	Potentially less severe/very severe	P(E)	birth	12 days	19 days	NA	No	No	1
12/M	c.[236_246del]; [1655 T > C]	Very severe/potentially less severe	P(E)	2	3	4	NA	No	Yes	1
13/F	c.[236_246del];[1927G > A]	Very severe/potentially less severe	P	6	11	11	NA	No	No	5^c^
14/M	c.[236-246del]; [236-246del]	Very severe	Neg	4	4	4	1:204.800	Yes prophylactic and later 1 cycle therapeutic [8]	Yes	1
15/F	c.[955 + 1G > A]; [1438-2A > G]	Unknown/very severe	NA	birth	5 days	8 days	≤ 1:400	No	No	1
16/M	c.[525delT]; [2237G > A]	Very severe	Neg (E)	birth	3	4	1:102.400	Yes 4 cycles at 1–2–3-4 years [8]	Yes	6^c^
17/M	c.[1A > G];[1A > G]	Very severe	Neg(E)	2	6	6	________NA	No	No	1
18/M	c.[784G > A]; [784G > A]	Potentially less severe	P	1	1.5	4	≤ 1:400	No	No	4^c^
19/M	c.[1802C > G]; [2800-1G > C]	Potentially less severe/unknown	P(E)	10 days	3	3	≤ 1:400	No	No	2^c^
20/F	c.[1564C > G];[1564C > G]	Potentially less severe	P(E)	4	4	5	1:1.600	No	No	1
21/F	c.[930_932delGTT];[1927G > A]	Unknown/ Potentially less severe	P(E)	birth	19 days	1	NA	No	Yes	**7** ^c^
22/F	c. [1927G > A];[1927G > A]	Potentially less severe	P(E)	4	4	5	NA	No	No	1
23/F	c.[1933 G > A];[1933 G > A]	Potentially less severe	P(E)	birth	1	1	NA	No	No	1
24/F	c.[1933G > A]; [1564C > G]	Potentially less severe/potentially less severe	P(E)	2	3	4	NA	No	No	1
25/F	c.[784G > A]; [1822C > T]	Potentially less severe/very severe	P	2	3	4	≤ 1:400	No	No	2^c^
26/F	c.[1064 T > C]; [2041-2A > C]	Potentially less severe/very severe	P(E)	3	5	8	≤ 1:400	No	No	3^c^
27/M	c.[1465G > A];[40_47del8]	potentially less severe/unknown	P(E)	birth	3	4	≤ 1:400	No	No	2^d^
28/M	c.[2237G > A];[1655 T > C]	Very severe/potentially less severe	P(E)	2	4	9	1:3200	No	Yes	2^d^

^a^As reported in https://www.erasmusmc.nl/klinische_genetica/research/lijnen/pompe_center E = estimated on genotype [23]; ERT dosing: 1 = 20 mg/kg/14 days, 2 = 40 mg/kg/14 days, 3 = 20 mg/kg/10 days, 4 = 40 mg /kg/10 days, 5 = 20 mg/kg/7 days, 6 = 40 mg /kg/7 days, 7 = 15 mg/kg/7 days; F female; IAR infusion associated reaction; M male; N negative; NA not available; P positive

^b^months, unless differently indicated

^c^Patients in whom the dosage of ERT was modified in course of follow up due to poor clinical outcome or to infusion associated reactions. Dosing at the beginning of ERT = 20 mg/kg/14 days

^d^ dosage of ERT received from the beginning of treatment

E = estimated on genotype [23]; ERT dosing: 1 = 20 mg/kg/14 days, 2 = 40 mg/kg/14 days, 3 = 20 mg/kg/10 days, 4 = 40mg /kg/10 days, 5 = 20 mg/kg/7 days, 6 = 40mg /kg/7 days, 7 = 15 mg/kg/7 days; F = female; IAR = Infusion Associated Reaction; M = male; N = negative; NA = not available

### Statistics

Overall survival (OS) was calculated as the time from birth to death for any cause; ventilator-free survival (VFS) as the time from birth to invasive ventilation or death. Observation periods were censored at the date of last contact when no event was observed. Patients were followed-up on ERT for a median time of 71 months (range 1.9–134.3).

OS and VFS curves were computed with the Cox regression model allowing for delayed entry, as all patients entered the study at ERT commencement. Analysis was performed overall and in subgroups defined by potential prognostic factors, such as CRIM status and age at start of ERT (≤ 3 months vs ≥ 3 months). Cumulative incidences of cardiac normalization and independent walking were calculated allowing for delayed entry, according to Andersen et al. [22]. All tests were two-sided. Analyses were performed with SAS 9.2.

## Results

Table 1 shows demographics, genetics and clinical data before ERT start as well as therapeutic management (ERT dosage and immunomodulation approach) and occurrence of infusion associated reactions (IAR) related to each patient. Median age at symptoms onset was 2 months (range 0–6), at diagnosis 3 months (range 5 days-11 months) and 4 months at ERT starting (range 8 days-11 months). The 17 patients who were alive at the end of data collection had been on ERT for a median of 71 months (range 25–134 months).

Residual GAA activity was less than 2% of the normal values for the reference laboratory in all patients (data not shown) and gene analysis was performed in 25 out of the 28 patients. CRIM status was tested on bloodspot or cultured fibroblasts by Western Blot analysis (lab. Great Ormond Street Hospital, London) in 5 patients and indirectly deduced from genotype [23] in other 19 patients. Seventeen of them (70.8%) were CRIM-positive and 7 (29.2%) CRIM-negative, while CRIM status was not available nor deducible in 4 patients.

Both at diagnosis and at ERT start all the patients showed: muscle weakness and hypotonia, increased serum CK (2 to 10 fold over the normal value) and severe hypertrophic cardiomyopathy, with increased thickness of septum and left ventricular wall. Eight out of the 28 patients (28.6%) had respiratory distress and one needed respiratory support (patient 16).

### Follow-up

The data on clinical follow-up are reported in Table 2. Nine patients (patients 1 to 9; 6 females, 3 males) died within the first 20 months of life (mean 13; median 15; range 5–20 months), 3 were CRIM positive (patients 3, 6, 8) and 3 CRIM negative (patients 5, 7, 9), while for 3 of them (patients 1, 2, 4) CRIM status was neither available nor estimated on genotype.

**Table 2:** Follow-up data of 28 IOPD patients treated with ERT

Patient ID/gender	CRIM	Survival (A or D)	Age last visit*	Cause of death	Asssisted ventilation (T or NIV or N)	Artificial nutrition (G or NG or N)	Motor achievements (none/HC/S/W)	Hearing deficit/intelligible speech	Heart normalization	Contractures at last visit
1/M	NA	D	5 m	2	N	N	none	na/na	partially	N
2/F	NA	D	6 m	3	N	N	none	na/na	partially	N
3/F	P(E)	D	7 m	2	N	N	none	na/na	partially	Y
4/M	NA	D	13 m	2	N	N	none	na/na	partially	N
5/F	Neg(E)	D	15 m	3	N	N	none	na/na	Y	N
6/F	P(E)	D	15 m	2	N	N	none	na/na	partially	N
7/F	Neg(E)	D	18 m	2	N	N	none	na/na	partially	N
8/F	P(E)	D	19 m	2	T	N	none	na/na	partially	N
9/M	Neg	D	20 m	1	N	N	S	N/N	partially	N
10/M	Neg (E)	D	5.0	1	T	G	none	na/N	partially	Y
11/M	P(E)	D	6.8	1	N	N	W	N/Y	Y	Y
12/M	P(E)	A	2.5		N	N	W	N/Y	Y	N
13/M	P	A	3.0		T	G	None (previously S)	Y/N	partially	N
14/F	Neg	A	2.5		NIV (2y)	NG	S	Y/N	partially	N
15/F	NA	A	4.0		N	N	W	N/Y	Y	Y
16/M	Neg (E)	A	4.0		T (3y)	G	None (previously S)	N/N	partially	Y
17/M	Neg(E)	A	4,4		T	G	none	N/N	Y	N
18/M	P	A	4.5		NIV	N	None (previously S)	N/N	Y	N
19/M	P(E)	A	5,8		T (5y)	G	S	N/N	Y	Y
20F	P(E)	A	6.5		T	G	HC (previously S)	Y/N	partially	N
21/F	P(E)	A	6.0		T	G	S	Y/N	Y	Y
22/F	P(E)	A	7.0		T	G	S	N/Y	Y	Y
23/F	P(E)	A	7.0		N	N	W	Y/Y	Y	N
24/F	P(E)	A	9.75		N	N	W	Y/Y	Y	N
25/F	P	A	9. 16		T (7y)	N	S	N/Y	Y	Y
26/F	P(E)	A	10.9		NIV (8.5 y)	N	S (previously W)	N/Y	Y	Y
27/M	P(E)	A	11.5		N	N	W	Y/Y	Y	Y
28/M	P(E)	A	11.5		T	G	none	Y/N	Y	Y

*years unless differently indicated; A alive, D dead, E: estimated on genotype [23], F female, G gastrostomy, HC head control, M : male, m months, N no, na not assessed, NA: not available, Neg: negative, NG nasogastric tube, NIV non invasive ventilation, P: positive,  S sitting independently, T tracheostomy, W walking independently, y:years, Y yes

Cause of death: 1- cardiorespiratory failure, 2- ERT withdrawn after failure to respond**,** 3- infusion-related reactions prevented ERT delivery

Concerning the “long-term” follow-up group, 14 patients resulted CRIM-positive (patients 11–13, 18–28), 4 CRIM negative (patients 10, 14, 16, 17) and one neither examined nor deducible from genotype (patient 15). Patients 10 and 11 died at the age of 60 and 78 months respectively, due to respiratory failure secondary to pneumonia. The current median age of the 17 surviving patients at time of data acquisition was 6 years (range 2–11.5 years).

### Ventilation

**Fig. 1:**
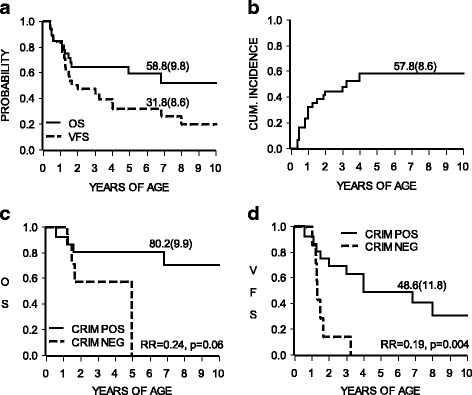
**a-** Overall survival (OS) and ventilation free survival (VFS) of the whole cohort of IOPD patients: at 6 years of age 58.8% (SE 9.8) and 31.8% (SE 8.6) respectively. **b-** overall cumulative incidence of cardiac normalization: at 6 years 57.8% (SE 8.6).  **c-** OS of CRIM-negative (NEG) and CRIM-positive (POS). **d**- ventilation free survival of CRIM-negative and CRIM-positive. In panel **c** and **d** the relative risk (RR) of failure for CRIM-positive vs. negative is reported, together with the p-value. CRIM positives’ risk of death was 1/4 of CRIM negative patients and the risk of being ventilated was 1/5 of that of CRIM negatives

At the last clinical examination, out of the 19 long-term surviving patients only 6, 31%, (patients 11, 12, 15, 23, 24, 27) were free of any ventilation support, 3 patients, 15%, (patients 14, 18, 26), needed non-invasive ventilation (NIV) and 10 patients, 52%, (patients 10, 13, 16, 17, 19–22, 25, 28) were ventilated through tracheostomy. Figure 1a shows the OS and the VFS for the whole cohort. Proportions of OS and VFS were 64.2% (SE 9.3) and 47.3% (SE 9.3) respectively at the age of 3 years and 58.8% (SE 9.8) and 31.8% (SE 8.6), respectively at the age of 6 years (individual data of survival and ventilation are reported in Table 2).

### Feeding

Nine (32%) of the long-term surviving patients (patients 11, 12, 15, 18, 23–27) maintained autonomous feeding capacities, including 2 patients receiving ventilation support (one tracheostomised). Nine patients (patients 10, 13, 16, 17, 19–22, 28) had gastrostomy and one (patient 14) was nourished by naso-gastric tube (Table 2).

### Motor function

**Fig. 2:**
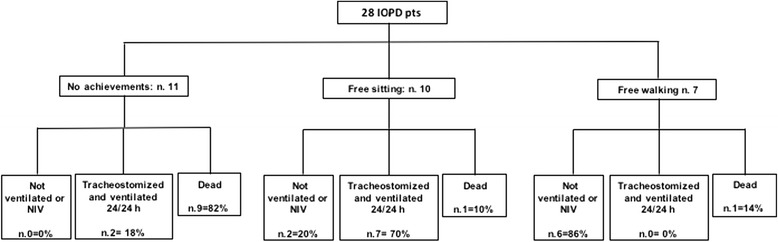
Outcome of 28 Italian IOPD patients in relation to the best motor milestone achieved

Only 7 (25%) patients (patients 11, 12, 15, 23, 24, 26, 27), reached independent ambulation at a median age of 16.5 months (range 12–19 months) and the overall cumulative incidence of independent ambulation was 23.7% (SE 7.6). All of them maintained the walking capacity except one (patient 26), who lost it at the age of 8.5 years due to progressive muscle weakness. The sitting position was achieved by 17/28 patients (60%; patients 9, 11–16, 18–27), although 4 (patients 13, 16, 18, 20) subsequently lost this skill (Table 2). One patient (patient 9) belonged to the group of patients who died before 20 months of life and was CRIM-negative. Figure 2 shows for the whole group of patients the relation to OS and VFS of the best motor milestone achieved.

At the time of last clinical evaluation, joint contractures (more frequent at lower limbs) were evident in 12 patients (Table 2). Facial muscle weakness and/or speech disorders and/or dysphagia were observed in all patients.

Nine patients (patients 11, 12, 15, 22–27) showed a hypernasal speech with intelligible language at an age ranging from 2.5 to 11.5 years. In the other patients language was unintelligible due to reduced movement of lip and tongue and velopharyngeal incompetence. Swallowing function was not routinely studied.

### Cardiac function

Heart parameters (septum with left ventricular wall thickness and ejection fraction) normalized in 15/28 patients (patients 5, 11, 12, 15, 17–19, 21–28) at a median age of 12 months (range 5–48 months) after a median treatment of 11 months (range 1–42 months). The other 13 patients (patients 1–4, 6–10, 13, 14, 16, 20) showed only a partial cardiac improvement (Table 2); of this group, 4 patients (patients 13, 14, 16, 20) were alive at a median age of 3.5 years (range 2.5–6 years) while 9 (patients 1–4, 6–10) died at a median age of 14 months (range 5–60 months). In Fig. 1b the estimated cumulative incidence of cardiac normalization at 6 years is reported: 57.8% (SE 8.6).

### Hearing function

Nineteen patients were formally tested with administration of behavioral audiometry or evoked potentials: 11 had no hearing defect (patients 9, 11, 12, 15–19, 22, 25, 26) and 8 (patients 13, 14, 20, 21, 23, 24, 27, 28; all CRIM-positive) showed different degrees of hearing deficit (Table 2). Nine patients have never been formally tested but were reported to apparently hear sounds included in the vocal extension (80–1500 Hz).

### Brain MRI abnormalities

**Fig. 3:**
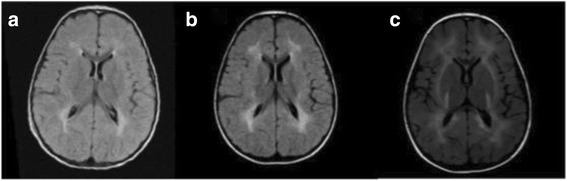
Axial section T2 flair at the ages of 2 (**a)**, 3 (**b)**, 6 (**c)** years: white matter is progressively affected. In the last image the damage is extended to the posterior limb of the internal capsule and the subcortical white matter with U fibers. Basal ganglia are spared

Brain MRI was performed in 6 CRIM-positive subjects (patients 11, 15, 19, 20, 23, 27). In one patient (patient 27) it was repeated at 2, 3 and 6 years of age, whereas in the other 5 the exam was performed only once at the age of 6 years. Imaging data showed the presence of moderate periventricular white matter abnormalities (hypomyelination) in all of them. Additionally, patient 27 showed a progressive deterioration of MRI parameters (Fig. 3)**,** associated with the worsening of cognitive performances (Wechsler scales IQ: WPPSI 85 at 3 years; 75 at 5 years 10 months; WISC III 73 at 8 years, 64 at 9 years, 50 at 11 years).

### Outcome according to CRIM status

Compared to the 7 CRIM-negative the 17 CRIM-positive patients showed a better outcome in terms of OS and VFS: risk of death in CRIM-positive patients was 1/4 compared to CRIM-negative patients (relative risk, RR = 0.24, 95% CI 0.05–1.10; *p*-value = 0.06) (Fig. 1c), while VFS risk (Fig. 1d) in CRIM-positive was 1/5 compared to CRIM-negative patients (RR 0.19, 95% confidence interval (CI) 0.06–0.59; p-value = 0.004). At the end of the study 13 CRIM-positive patients were alive at a median age of 7 years (range 2.5-11.5). Twelve out of 17 CRIM-positive patients (patients 11, 12, 18, 19, 21–28) achieved normalization of heart function within 6 years of age, with a 6 year cumulative incidence of 74.2% (SE 9.4). On the contrary, only 2/7 (28%) CRIM-negative patients (patients 5 and 17) normalized their cardiac parameters.

Of the 7 infants who reached independent ambulation 6 were CRIM-positive (patients 11, 12, 23, 24, 27; 35% of CRIM-positive patients) and one had unknown CRIM status (patient 15) and very low-titer antibodies (≤1:400). No CRIM-negative patients achieved walking ability.

### Outcome according to age at starting ERT

The effect of age at ERT starting (≤ 3 months and ≥3 months) on OS, VFS, normalization of cardiac parameters, and achievement of independent ambulation, was analysed on the whole cohort and no significant differences were found. Within the CRIM-positive subgroup, only 4 patients had started ERT ≤ 3 months of age (patients 11, 19, 21, 23). This limited number prevented further investigations on the effect of an early start of ERT in the CRIM-positive subgroup.

### Anti-rhGAA antibodies, adverse events, and immunomodulation treatment

Data concerning the titers of anti-rh-GAA antibodies were available in 13/28 (46%) patients (7 CRIM-positive: patients 18–20, 25–28; 5 CRIM-negative: patients 5, 9, 10, 14, 16; one unknown: patient 15) (Table 1). The **7** CRIM-positive patients and the unknown one had low-titer antibodies (≤ 1:3200), while the 5 CRIM-negative had intermediate to high antibody titers, with maximum values ranging from 1:25.600 to 1: 204.800.

Eight patients (3 CRIM-positive: patients 12, 21, 28; 4 CRIM-negative: patients 5, 10, 14, 16; one unknown: patient 2) experienced IAR  (flushing, urticaria, bradycardia, respiratory distress).

Three of them (patients 12, 14, 28) were successfully treated with a premedication protocol including the use of corticosteroids or antihistaminics and decreased infusion rate. Two patients (16 and 21) who presented very severe reactions (massive urticaria and anaphylactic shock with glottis oedema respectively) followed a desensitization protocol. It initially provided a very diluted dose of ERT (1/10 or 1/20 of the recommended dilution administered in 24–48 h) and therefore a progressive increase in dosage and concentration of the enzyme over a period of 6 months. Other 2 CRIM-negative patients (2 and 5) never received any kind of desensitization treatment and ERT was interrupted. Patient 10 was immunomodulated at the time of IAR (see below).

Three CRIM-negative patients (patients 10,14,16) received a tolerance induction protocol, according to the described experiences [7, 8], 2 of them (patients 10, 16) after having developed an anti-rhGAA antibodies titer > 1: 51.200 and one (patient 14) on a prophylactic basis, simultaneously with the first dose of rhGAA.

During follow-up period, all 3 patients showed a progressive loss of the motor, cardiac and ventilatory monitored functions. Patient 10 died at the age of 60 months after having received one cycle of immunomodulation. Due to periodic increase of anti-rhGAA antibodies, patient 16 has received 4 cycles of therapeutic immunosuppression [8] and has then changed the immunomodulation protocol [9]. Patient 14, who received prophylactic treatment, showed an increase of anti-rh-GAA to 1:204.800 after few months of ERT and received a cycle of therapeutic immunosuppression protocol [8]. One year after, antibodies titer increased again to 1:102.400 in concomitance to a very poor clinical condition (Table 2) and immunotherapy was not repeated.

## Discussion

Our study presents the results of a retrospective analysis of the clinical outcome of 28 Italian IOPD patients receiving ERT. The studied cohort included 17 CRIM-positive, 7 CRIM-negative and 4 not CRIM-defined patients who were followed for a median period of 6 years (range 2.5–11.5 years) in 13 Italian reference centres. To our knowledge this is the longest independent follow-up study in a heterogeneous group of IOPD patients. As reported in Table 2 and shown in Fig. 1, the survival data analysis confirmed the poorest prognosis of CRIM-negative patients, with only 4  surviving beyond 2 years of age (one of them, patient 10, died at 5 years), while at the end of the study 13 CRIM-positive children were alive at a median age of 7 years.

The Kaplan Meier survival rate of our patients was 64.2% (SE 9.3) at the age of 3 years and 58.8% (SE 9.8) at 6 years of age; the survival without invasive ventilation was 47.3% (SE 9.3) at 3 years and 31.8% (SE 8.6) at 6 years (Fig. 1). These results indicate a progressive worsening of the whole group of patients with age. They also are in agreement with those of other long-term ERT follow-up studies that reported a survival rate varying from 54% to 72% and a VFS of 35% to 40% [12, 13, 15]. In particular, they are quite similar to those reported by Kishnani et al. [3] in the phase III extension study at 3 years of age: rate of survival 72% and rate of survival without ventilation 49%.

Taking into account the achievement of developmental milestones, independent ambulation was reached by 25% of our patients, a percentage consistent with the data of 20 to 50% described by other authors [12, 14]. As expected and already observed by others [13] the ERT effects on motor development paralleled those on OS and VFS (Fig. 2). Also the therapeutic response of monitored cardiac parameters (left ventricular wall thickness and ejection fraction), which normalized at a median age of 6 years in 57.8% of our patients (Fig. 1), was in agreement with the results observed by Hahn et al. and Broomfield et al. [13, 15]. In fact, they showed normalization of heart parameters in 52% and 73% of their cohorts, respectively. Similar percentages were obtained for the need of assisted feeding that was necessary in 52% of our patients, while in other long-term studies it varied from 40 to 65% [12, 15].

Analysing the presence of hearing loss, we observed this complication in 57% of our patients, data consistent with literature results [24, 25]. In contrast with these observations, Hahn et al. described a hearing impairment in only 3 out of the 23 patients of their casuistry [13].

A worse therapeutic response to ERT of CRIM-negative patients has already been described by other long-term follow-up experiences [13–15]. In fact, Broomfield et al. [15] reports that only 33% of the CRIM-negative patients survived up to 42 months of life in comparison with a survival rate of 85% in the CRIM-positive group. Even less positive data emerged from the studies of Hahn et al. [13] and van Gelder et al. [14], who reported few CRIM-negative patients surviving until the age of 24 months. Finally, in the retrospective review aimed to analyse the emerging phenotype of long-term survivals (≥ 5 years of age), Prater et al. found that all CRIM-negative patients died before reaching the age of 5 years and therefore no CRIM-negative patient could be included in the study [26].

Our data are in line with these previous findings. Indeed, none of the 4 long-term surviving CRIM-negative patients maintained neither autonomous ventilator capacity nor oral feeding, nor reached independent walking or intelligible speech (Table 2).

The poorer prognosis of CRIM-negative patients has been mainly associated with the presence of elevated anti-rhGAA antibodies titers [5, 14, 27]. In our study, antibody testing was available only for 13/28 patients and also in our experience higher anti-rhGAA antibodies titers, varying from 1:25.600 to 1:204.800, were associated with the CRIM-negative status.

In an attempt to inhibit the development of antibodies in the CRIM-negative patients, immunotherapy was proposed by several authors [6–9]. These protocols demonstrated to be effective in reducing the antibody level and in inhibiting their production for long time. Furthermore, immunomodulatory treatment started simultaneously with ERT showed to be more effective than immunosuppression in patients who had already developed a significant anti-rhGAA response [8, 9]. However only one patient of our study was treated with the pre-emptive protocol but without any apparent long-term efficacy in controlling the antibody response.

In agreement with the literature [12–15], we have confirmed the better prognosis of CRIM-positive patients. However, ventilation, feeding and muscular function data showed clear clinical deterioration in at least 8 CRIM-positive patients, including those who had responded positively to an early ERT **(**Table 2). A similar long-term outcome, with a progressive impairment of pulmonary and muscle functions in CRIM-positive patients has been already described [12–15].

Recently, some authors suggested the efficacy of an increased ERT dosage in improving muscular outcome and reducing respiratory events that require hospitalization [16, 17]. Moreover, a dose-dependent effect of ERT in improving intracellular clearance and reducing the glycogen storage in skeletal and heart muscles has been described in vitro and in animal models [28–30].

The development of neonatal screening programs will probably modify the future of IOPD patients. The Taiwan experience showed that a very early diagnosis with a consequent pre-symptomatic ERT start, resulted in a better prognosis [10, 11]. In particular, Yang et al. [11] showed that even few days of difference in therapy start (mean of 12 vs 21 days) may play a significant role in the clinical outcome. After one year of follow-up, they demonstrated a better improvement of biochemical parameters and functional tests in the very early treated CRIM-positive patients. These results, compared to those of CRIM-positive patients who begun therapy at symptoms appearance [12–15], suggest a positive impact of newborn screening programs for Pompe disease. However, an increasing number of observations have been recently published showing that early pre-symptomatic treatment in CRIM-positive, low antibody titer patients does not completely prevent slow deterioration after the first years of life [10, 31, 32]. Moreover pre-symptomatic treatment does not always guarantee a positive outcome in a short-term, as shown by Schänzer et al. [33], who reported an extreme case of a CRIM-positive, low antibody titer IOPD infant, treated since the 3rd day of life with 40 mg/kg/week who never achieved free sitting position and was tracheostomized and ventilated from 10 months on [33].

Prolonged follow-up studies are necessary. The retrospective-observational design of our study and the limited number of CRIM-positive patients receiving early ERT did not allow us to define any relation between clinical outcome and age of therapy start.

Finally, an emerging finding in IOPD patients is the presence of periventricular white matter abnormalities [18–21]. We detected a pattern of hypomyelination in 6 children who underwent brain MRI. In one of them the exam was repeated 3 times during follow-up showing a progressive worsening of the lesions paralleling the development of a progressive cognitive impairment. A similar evolution has also been described by Ebbink et al. in other patients [19, 20]. These radiological abnormalities might be caused by neuronal glycogen storage [18] and the inability of the enzyme to cross the blood-brain barrier [28, 30] may justify the progressive cognitive and psychomotor worsening observed in these patients.

## Conclusions

In conclusion, after 10 years of ERT we are aware of the long-term poor outcome of CRIM-negative patients but at the same time it has emerged that many CRIM-positive patients, in spite of an initial ERT positive response fail to improve or stabilize their clinical conditions. The development of neonatal screening programs, allowing a very early pre-symptomatic beginning of ERT could lead to a significant improvement of the clinical outcome. However, long-term studies are still lacking. Furthermore, to improve treatment efficacy, shared protocols of immunomodulation and ERT high dosage/frequent infusion trials in a wider number of patients, are needed.

Finally, brain involvement in Pompe disease remains an open issue not treatable by ERT in the current formulation and represents one of the main goals to be pursued by future research studies.

## Abbreviations

CI: Confidence interval
CRIM: Cross reactive immunologic material
eow: Every other week
ERT: Enzyme Replacement Therapy
GAA: Acid alpha glucosidase
IAR: Infusion Associated Reaction
IgG: Immunoglobulin G
IOPD: Infantile Onset Pompe Disease
MRI: Magnetic Resonance Imaging
NIV: Non-invasive ventilation
OS: Overall Survival
rhGAA: Recombinant human acid α-glucosidase
RR: Relative risk
SE: Standard error
VFS: Ventilator free survival
WISC III: Wechsler intelligence scale for children -third edition
WPPSI: Wechsler Preschool and Primary Scale of Intelligence
